# Sprayable and biodegradable, intrinsically adhesive wound dressing with antimicrobial properties

**DOI:** 10.1002/btm2.10149

**Published:** 2019-12-13

**Authors:** John L. Daristotle, Lung W. Lau, Metecan Erdi, Joseph Hunter, Albert Djoum, Priya Srinivasan, Xiaofang Wu, Mousumi Basu, Omar B. Ayyub, Anthony D. Sandler, Peter Kofinas

**Affiliations:** ^1^ Fischell Department of Bioengineering University of Maryland College Park Maryland; ^2^ Sheikh Zayed Institute for Pediatric Surgical Innovation Joseph E. Robert Jr. Center for Surgical Care, Children's National Medical Center Washington District of Columbia; ^3^ Department of Chemical and Biomolecular Engineering University of Maryland College Park Maryland; ^4^ Department of Chemistry and Biochemistry University of Maryland College Park Maryland

**Keywords:** antimicrobial wound dressings, biodegradable polymers, silver, solution blow spinning, wound healing

## Abstract

Conventional wound dressings are difficult to apply to large total body surface area (TBSA) wounds, as they typically are prefabricated, require a layer of adhesive coating for fixation, and need frequent replacement for entrapped exudate. Large TBSA wounds as well as orthopedic trauma and low‐resource surgery also have a high risk of infection. In this report, a sprayable and intrinsically adhesive wound dressing loaded with antimicrobial silver is investigated that provides personalized fabrication with minimal patient contact. The dressing is composed of adhesive and biodegradable poly(lactic‐*co*‐glycolic acid) and poly(ethylene glycol) (PLGA/PEG) blend fibers with or without silver salt (AgNO_3_). in vitro studies demonstrate that the PLGA/PEG/Ag dressing has antimicrobial properties and low cytotoxicity, with antimicrobial silver controllably released over 7–14 days. In a porcine partial‐thickness wound model, the wounds treated with both antimicrobial and nonantimicrobial PLGA/PEG dressings heal at rates similar to those of the clinical, thin film polyurethane wound dressing, with similar scarring. However, PLGA/PEG adds a number of features beneficial for wound healing: greater exudate absorption, integration into the wound, a 25% reduction in dressing changes, and tissue regeneration with greater vascularization. There is also modest improvement in epidermis thickness compared to the control wound dressing.

## 1 INTRODUCTION

Infections are a substantial yet preventable cause of morbidity. For example, patients with burn wounds covering greater than 40% total body surface area (TBSA) have a 75% risk of mortality due to infection.^1^ Orthopedic trauma and surgical wounds in low resource settings also have an extraordinarily high risk of infection.^2^, ^3^ Exposure to infectious agents can cause sepsis and may contribute to harmful immune responses.^4^, ^5^ A sprayable, biodegradable, and antimicrobial wound dressing may decrease morbidity by reducing contact during wound care, eliminating the need for frequent dressing changes, and delivering drugs that can reduce the risks of infection.

Conventional wound dressings—typically adhesive‐coated, nondegradable, and thin polymer films—are prefabricated and nonconformal, making them difficult to use on large TBSA wounds or traumatic injuries, which are irregular in shape and depth. To prevent hematoma formation and exudate buildup, these dressings often require frequent changes that are painful, disrupt the healing epidermis, and may increase the risk of infection.^6^, ^7^ Hydrogel dressings attempt to address these limitations by providing a moist wound healing environment that can be delivered as soft, moldable material.^8^, ^9^ When removal is necessary, they can be dissolved on‐demand by exchanging crosslinks.^10^ Nanofibrous wound dressings provide excellent absorption of wound exudate and oxygen permeation because of their high porosity, but are typically prefabricated using electrospinning.^11^, ^12^ Composites of both approaches have been explored to provide a combination of porous structure and moisture.^13^ However, no current wound dressing provides custom sprayable fabrication with no patient contact, exudate absorption, and adhesion to the wound without additional fixation.

Here, we investigate solution blow spinning (SBS) as a sprayable method for the direct deposition of biodegradable polymer fibers containing antimicrobial silver onto wounds. Unlike electrospinning, which uses an applied voltage to drive fiber production and has low production rates, SBS uses a pressurized gas to produce fibers from a polymer solution with high production rates.^14^ A polymer blend solution of poly(lactic‐*co*‐glycolic acid) and poly(ethylene glycol) (PLGA/PEG) sprayed with a portable airbrush, produces intrinsically adhesive polymer fibers that accumulate on and seal the wound. Silver salts are incorporated to create a sprayable and antimicrobial wound dressing (PLGA/PEG/Ag) that can release bactericidal silver ions and reduce the risk of infection. These commonly used silver salts have broad‐spectrum antimicrobial activity with relatively low minimum inhibitory concentrations (MIC) and minimum bactericidal concentrations (MBC).^15^, ^16^, ^17^, ^18^, ^19^, ^20^

In this report, we examined the effect of silver nitrate (AgNO_3_) on silver ion (Ag^+^) release, mechanical properties, and adhesion of PLGA/PEG. in vitro studies were used to determine the optimal concentration of AgNO_3_ loaded into PLGA/PEG spinning solutions. To demonstrate the feasibility of using PLGA/PEG and PLGA/PEG/Ag wound dressings, they were evaluated in an in vivo porcine partial‐thickness excisional wound model. The incorporation of biodegradable PLGA/PEG into the scab and its absorption of wound exudate were examined using histology and fluorescence microscopy. Dressing changes were made as needed and tracked to demonstrate the potential benefits of using an intrinsically adhesive dressing that is biodegradable and can be absorbed by the wound.

## 2 RESULTS

### 2.1 Effect of solvent on fiber morphology and silver content

We first evaluated whether solvent properties could affect the morphology of fibers produced by SBS, which is a critical factor in fiber formation. Acetone has been previously used for SBS processes and does not affect cell viability.^21^, ^22^ However, AgNO_3_'s solubility in acetone at room temperature is only 4.4 mg/mL.^23^ Ethyl acetate has lower toxicity than acetone and is able to dissolve nearly 10 times as much AgNO_3_, up to a concentration of 27 mg/mL.^24^ This makes it an excellent alternative solvent for SBS of polymers in situ.

**Figure 1:**
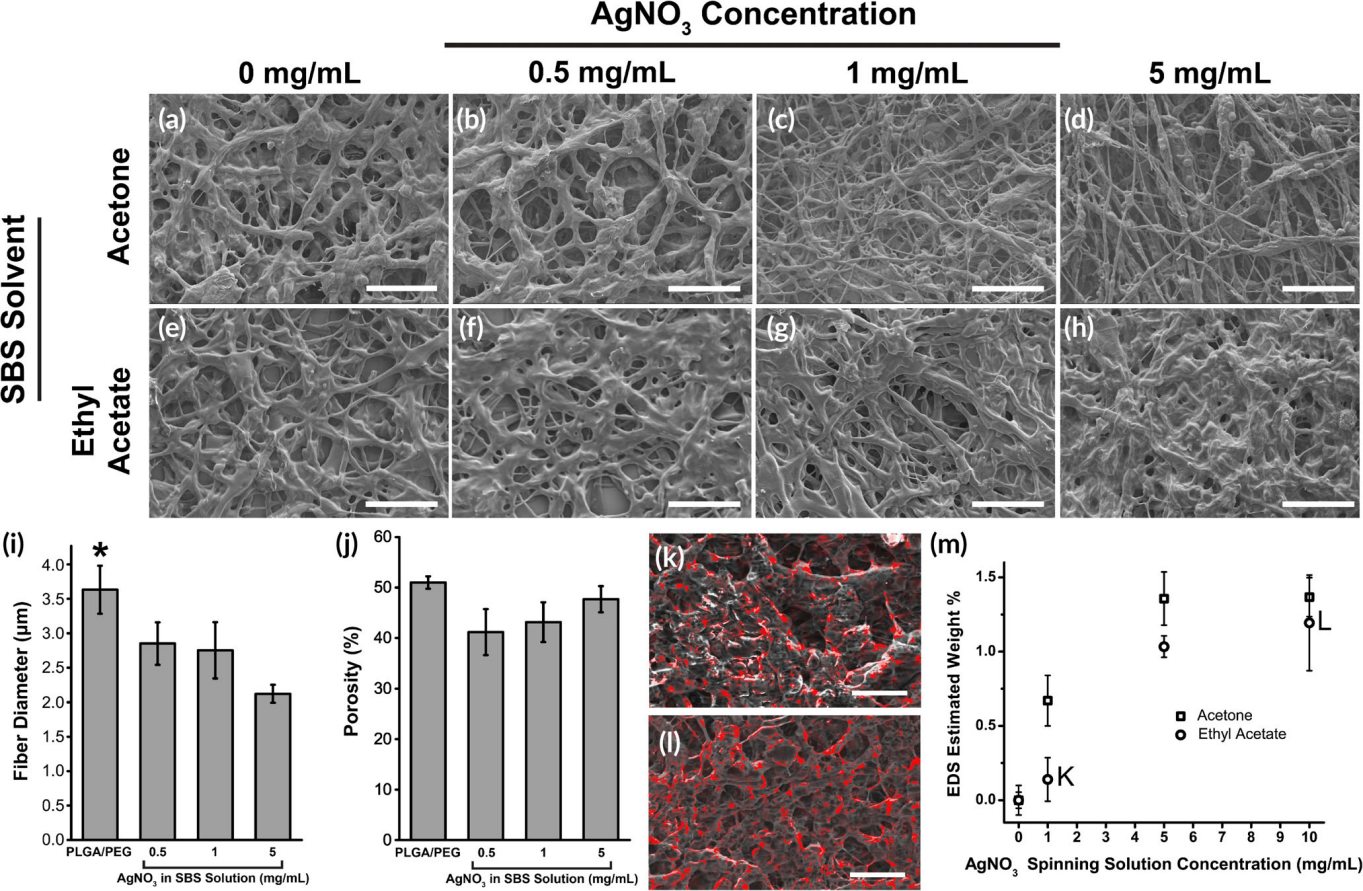
SEM images of PLGA/PEG fibers produced using solution blow spinning with increasing concentrations of AgNO_3_ added to the spinning solution. Fibers produced using acetone as the spinning solvent (a–d) create a beads‐on‐a‐string morphology when loaded with AgNO_3_, while those made with ethyl acetate (e–h) have a consistent web‐like fiber morphology. Scale bar = 100 μm. When using ethyl acetate as the spinning solvent, there are decreases in fiber diameter with AgNO_3_ concentration (i), while porosity (j) is similar (*n* = 2–4). Energy dispersive x‐ray spectroscopy (EDS) shows that PLGA/PEG fibers produced using solution blow spinning with ethyl acetate contains silver. EDS signal (red) superimposed on scanning electron microscopy images of fibers produced from polymer solution containing 1 mg/mL (k) and 10 mg/mL (l) AgNO_3_. Scale bar = 10 μm. (m) Plot of estimated weight percent of silver calculated from EDS for PLGA/PEG spinning solutions varying in AgNO_3_ concentration. Asterisks indicate statistical significance: *p < .05; **p < .01; ***p < .001

The pure PLGA/PEG samples for both acetone and ethyl acetate resemble each other in morphology (Figure 1a,e). However, SBS from acetone produces fibers that exhibit an AgNO_3_ dependent change towards a beads‐on‐a‐string morphology (Figure 1b–d), which is characteristic of polymer solution jets that form spherical structures during the spraying process.^25^ SBS from ethyl acetate continues to produce fibrous structures up to 5 mg/mL AgNO_3_ (Figure 1f–h); so, we therefore selected ethyl acetate as the spinning solvent for all of the following experiments. Using ethyl acetate, fiber diameter decreases as silver content increases (Figure 1i) while porosity remains constant (Figure 1j)_._ The difference in morphology may be related to the lower solubility of AgNO_3_ in acetone, which evaporates quickly during spraying and may cause the aggregation of the silver salt. Ethyl acetate solubilizes much higher concentrations of AgNO_3_ and has a lower evaporation rate than acetone, meaning that it is less likely to form such precipitates during spraying. The lower evaporation rate of ethyl acetate may also account for the high degree of branching or welding observed between fibers.

Energy‐dispersive X‐ray spectroscopy (EDS) was used to perform elemental analysis on blowspun samples of PLGA/PEG containing from 1 to 10 mg/mL of AgNO_3_ in solution. From three 40 × 40 μm images, as shown in Figure 1k–l with red color superimposed, we estimated the weight percent (wt%) of silver in blow spun fibers produced from three different PLGA/PEG/Ag solutions containing 1, 5, and 10 mg/mL of AgNO_3_. The measured wt% varies from 0.67 to 2.4% when sprayed from acetone and from 0.14 to 1.19% when sprayed from ethyl acetate (Figure 1m).

### 2.2 Thermal and mechanical properties

**Figure 2:**
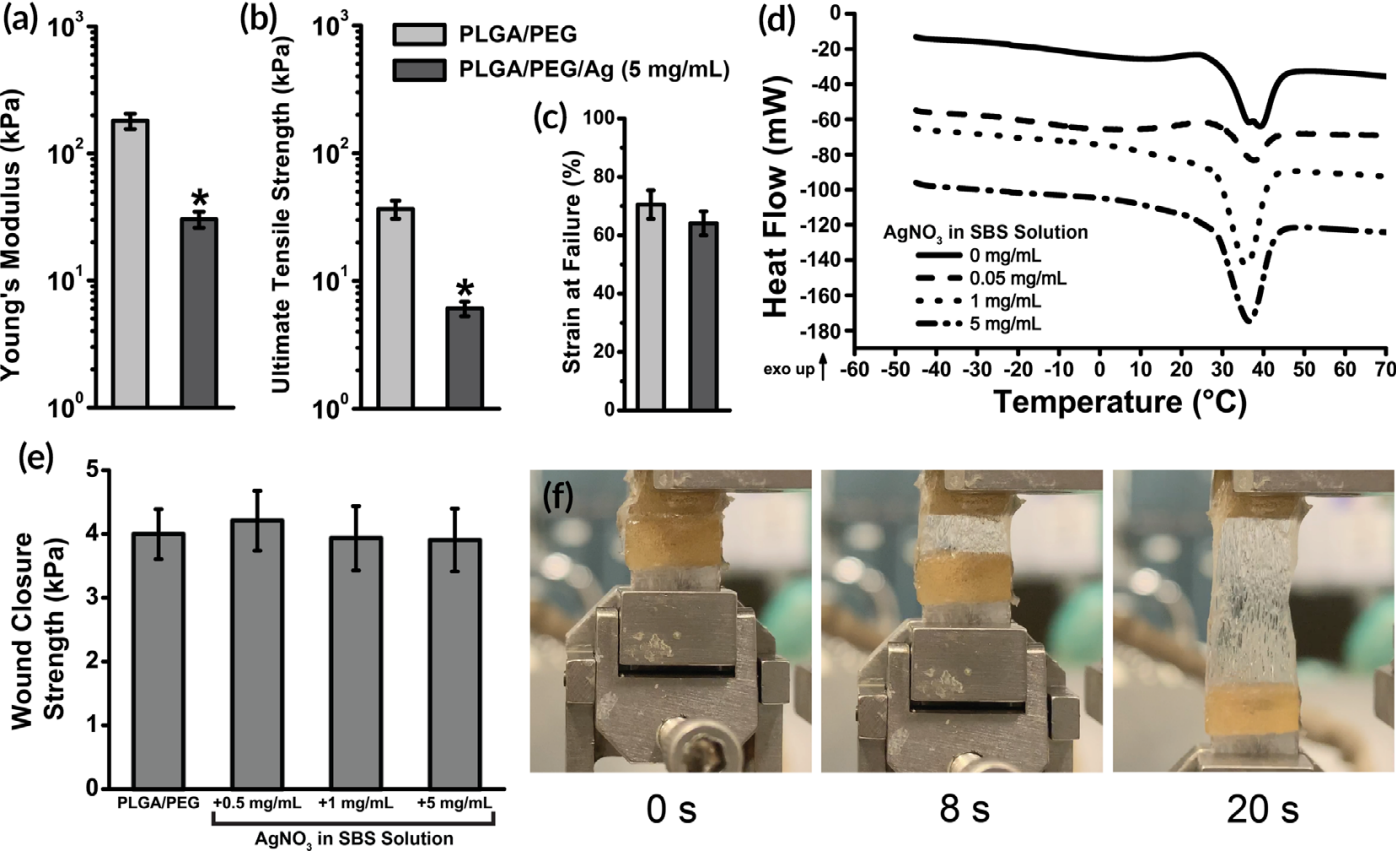
Mechanical testing of PLGA/PEG/Ag wound dressings (*n* = 5). Adding AgNO_3_ softens the wound dressing, producing lower Young's modulus (a), lower ultimate tensile strength (b), and comparable strain at failure (c). (d) All polymer blends with AgNO_3_ exhibit a body temperature mediated melting event at approximately 35°C using differential scanning calorimetry (DSC). Curves are shifted vertically for clarity. (e) Wound closure strength of PLGA/PEG/Ag dressings is constant as AgNO_3_ concentration increases (*n* = 5). (f) Images of PLGA/PEG/Ag (1 mg/mL) at 0, 8, and 20 s after the start of the wound closure strength test, showing the adhesive at high strain. Asterisks indicate statistical significance: *p < .05; **p < .01; ***p < .001

The effect of AgNO_3_ on the mechanical and thermal properties of PLGA/PEG was then evaluated. At the highest concentration tested (5 mg/mL), incorporating AgNO_3_ significantly decreases the tensile strength and stiffness of PLGA/PEG/Ag fiber mats. Young's modulus (Figure 2a) and ultimate tensile strength (Figure 2b) each decrease by approximately one order of magnitude, while strain at failure is unchanged (Figure 2c). Despite these decreases, our previous research has indicated that softer and more flexible polymer composites form a better interface with tissue and therefore can have greater adhesion strength and adhesion energy.^26^, ^27^, ^28^ PLGA/PEG undergoes a thermal transition while heating to body temperature triggered by PEG's melting, which allows the fibrous mat to become an intrinsically adhesive, conformal film. This transition is consistent regardless of silver content (Figure 2d). Wound closure strength was measured according to ASTM F2458–05 on porcine skin (Figure 2e), showing PLGA/PEG's combination of adhesion and cohesion (Figure 2f). Wound closure strength is unaffected by the addition of AgNO_3_.

### 2.3 Silver release kinetics

The release rate of Ag^+^ was studied by immersing samples of PLGA/PEG/Ag in 37°C water for up to 1 month. Periodically over 28 days, the supernatant was collected and analyzed using inductively coupled plasma‐atomic emission spectroscopy (ICP‐AES) to determine the concentration of Ag^+^. This study tested three different AgNO_3_ concentrations in polymer solution (0.5–5.0 mg/mL). The objective of this study was to determine which formulations released an appropriate amount of Ag^+^, guided by three benchmarks described in the literature: (a) The 24 hr cytotoxic limit, which is approximately 50 μM for fibroblasts,^29^, ^30^ (FCC) and higher for keratinocytes, 500 μM (KCC).^31^, ^32^ (b) The (MBC, which is approximately 20 μM for a small model bacterial inoculation.^33^ (c) The MIC, which is around 5 μM for an inoculation of the same size.^33^

**Figure 3:**
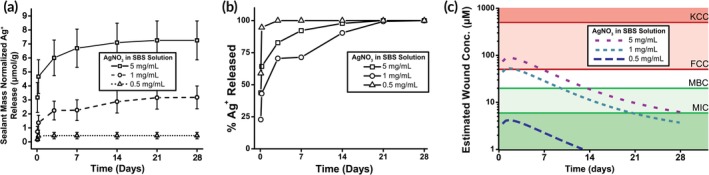
Silver ions can be released by solution blow spun (SBS) polymer fiber wound dressings over several days (*n* = 3). (a) Silver ions released in vitro are proportional to the concentration of silver in the SBS polymer solution. (b) The fraction of silver ions released over time varies based on how much silver is loaded into the wound dressing. (c) Estimated concentration in a wound based on the amount of silver released by a wound dressing produced using 2 mL of polymer solution. Data from (a) were fit to a logarithmic regression model and this was used to estimate concentration in the wound, accounting for projected first‐order absorption kinetics of Ag^+^

Depending on initial AgNO_3_ loading concentration, PLGA/PEG/Ag releases between 0.5 and 5 μmol per gram of wound dressing (Figure 3a). When loaded with more AgNO_3_, the release profile changes, with silver release extending over 7 days for 1 and 5 mg/mL PLGA/PEG/Ag (Figure 3b). Ag^+^ release is nearly complete within 1 day for PLGA/PEG/Ag with 0.5 mg/mL AgNO_3_. Higher AgNO_3_ concentrations had approximately 50% release at 1 day and reached 100% release after approximately 14 days. The differences in release kinetics are related to the size of the burst phase, which occurs during the first hours of drug release and typically accounts for a significant portion of drug release in PLGA devices.^34^, ^35^, ^36^

We estimated the Ag^+^ concentration in a partial‐thickness wound sprayed with 2 mL of polymer solution, given a penetration depth of Ag^+^ into the wound bed of 7.5 mm.^34^ Each data set was fit to a logarithmic model (Figure S1), which was then used to estimate absorption, distribution, metabolism, and excretion of Ag^+^ in the wound using previously described first order kinetics.^35^ This model, which combines in vitro Ag^+^ release data with realistic approximations for wound volume and various clearance rates, is shown in Figure 3c. With these design benchmarks, an AgNO_3_ concentration of 1 mg/mL in the SBS solution was selected that produces a concentration in the wound that is greater than the MBC, but does not greatly exceed the FCC. These SBS dressings could thus remain antimicrobial after application without strong cytotoxicity to the cells in the wound. This model likely overestimates the rate of Ag^+^ release due to the aqueous in vitro environment, which accelerates Ag^+^ release compared to the interface of the healing wound.

### 2.4 Antimicrobial activity and L929 cytotoxicity

**Figure 4:**
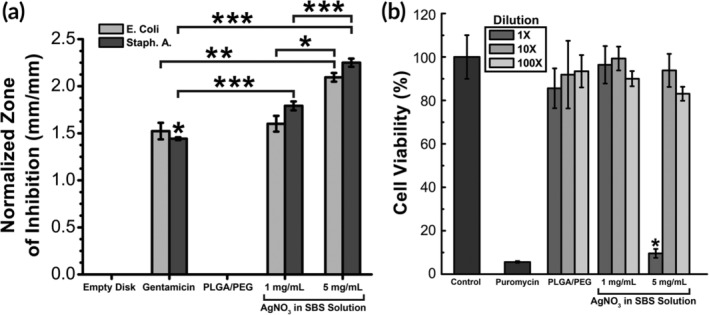
(a) Zone of Inhibition (ZOI) produced by PLGA/PEG with varying amounts of silver. Statistical significance was determined within bacteria type (*n* = 3–5). (b) Cytotoxicity of sealants to L929 mouse fibroblasts (*n* = 8). Moderate concentrations of AgNO_3_ (1 mg/mL) produce a high ZOI with no cytotoxicity. Asterisks indicate statistical significance: *p < .05; **p < .01; ***p < .001

Silver, while antimicrobial, can be cytotoxic at high concentrations. We aimed to determine a concentration of AgNO_3_ to incorporate into the polymer mats that suppresses bacterial growth with minimal toxicity. Bacterial growth inhibition was measured by the method of Kirby–Bauer disk diffusion (Figure 4a). This inhibition was tested using two commonly infectious Gram‐negative and Gram‐positive bacteria—*Escherichia coli* and *Staphylococcus aureus*. Concentrations greater than 1 mg/mL AgNO_3_ produced a normalized zone of inhibition (ZOI) that was not significantly different than the gentamicin control. A greater AgNO_3_ concentration of 5 mg/mL produced a larger ZOI, while PLGA/PEG alone produced no ZOI.

Cytotoxicity towards L929 mouse fibroblasts was tested at the antimicrobial concentrations of AgNO_3_ (Figure 4b). As shown, an AgNO_3_ concentration of 5 mg/mL led to significant cytotoxicity compared to 95% cell viability at 1 mg/mL. The optimal concentration of AgNO_3_ of 1 mg/mL produced antimicrobial efficacy yet also allowed for sufficient cell viability.

### 2.5 Porcine wound model

**Figure 5:**
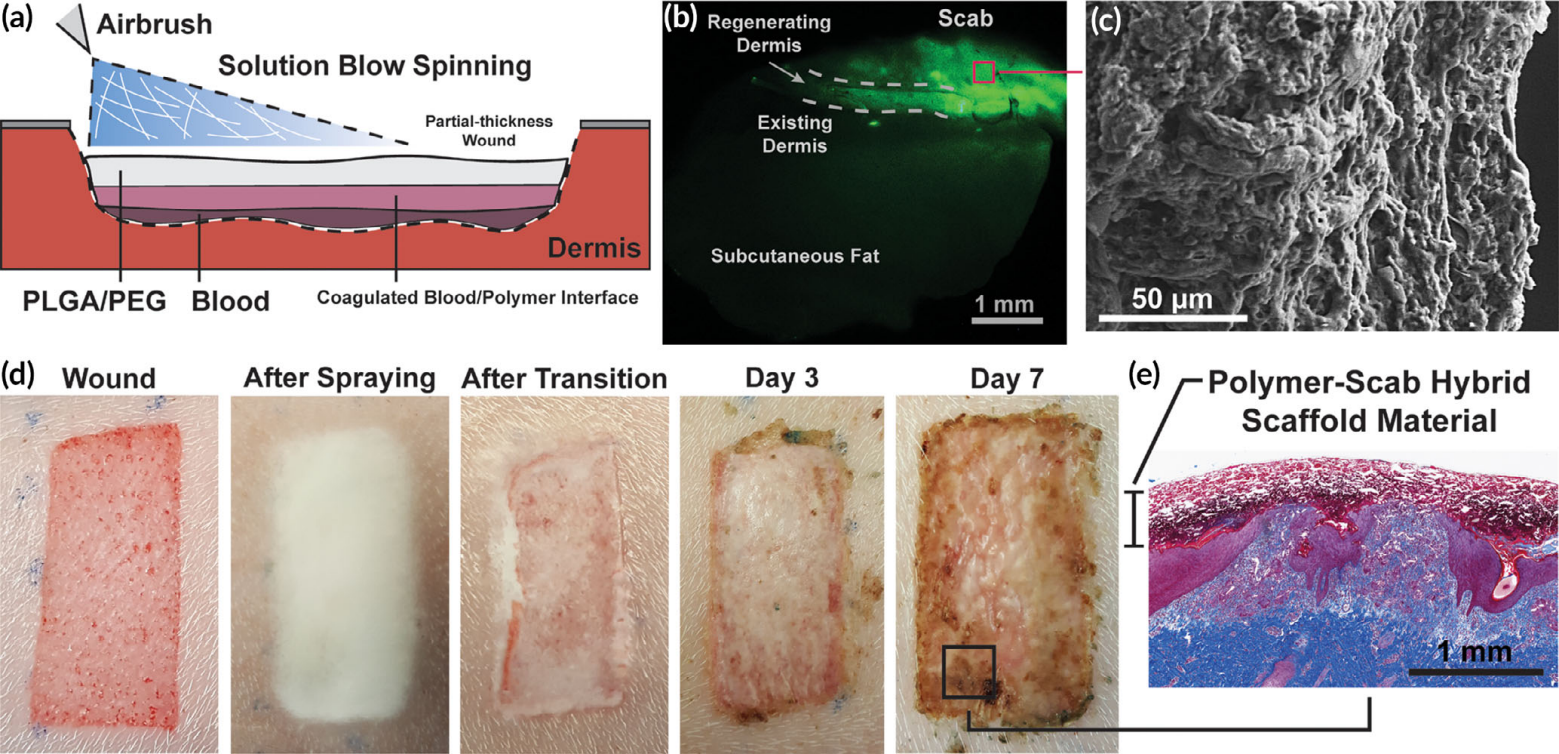
Schematic representation of solution blow spinning process for creating a PLGA/PEG wound dressing in situ. (a) Solution blow spun fibers can be deposited directly into a wound. (b) Fluorescent PLGA is integrated into the scab. (c) Scab cross section viewed using scanning electron microscopy. (d) Progression of wound dressing before and after adhesive thermal transition, and at 3 and 7 days after use. (e) Histological cross section of wound biopsy, stained with Masson's trichrome, showing the polymer‐scab hybrid scaffold material at day 7

Demonstration of efficacy in wound care requires evaluating the SBS dressings in comparison to commercially available controls. Direct deposition of PLGA/PEG and PLGA/PEG/Ag wound dressings by SBS was investigated in a porcine partial‐thickness wound model to characterize wound healing. The control dressing was Tegaderm, an adhesive‐coated, semipermeable polyurethane film often used in wound care specifically for skin harvest sites.^36^ Wounds were created with a Dermatome and were immediately dressed with either PLGA/PEG, PLGA/PEG/Ag (1 mg/mL AgNO_3_), or Tegaderm. In this model, PLGA/PEG and PLGA/PEG/Ag dressings were sprayed directly onto the wounds with an airbrush (Figure 5a). Spraying 2 mL of polymer solution uniformly across the wound produced complete wound coverage. A polymer‐scab composite material is formed as blood coagulates at the interface with the PLGA/PEG dressing, which was demonstrated utilizing fluorescent PLGA (Figure 5b). The resulting cross section of the entire scab at post wound day (PWD) 7 fluoresces, indicating that PLGA is incorporated throughout. Additionally, cross‐sectional SEM shows the microfibrous network of fibrin, exudate, and PLGA in detail (Figure 5c). PLGA/PEG initially adheres to the wound due to the adhesive thermal transition characterized in Figure 4d, where the porous SBS nanofiber mat becomes an adhesive thin film (Figure 5d). Biodegradable and absorbable PLGA/PEG is then incorporated into the wound, where it forms a durable scaffold and barrier throughout the re‐epithelialization process (Figure 5e).

**Figure 6:**
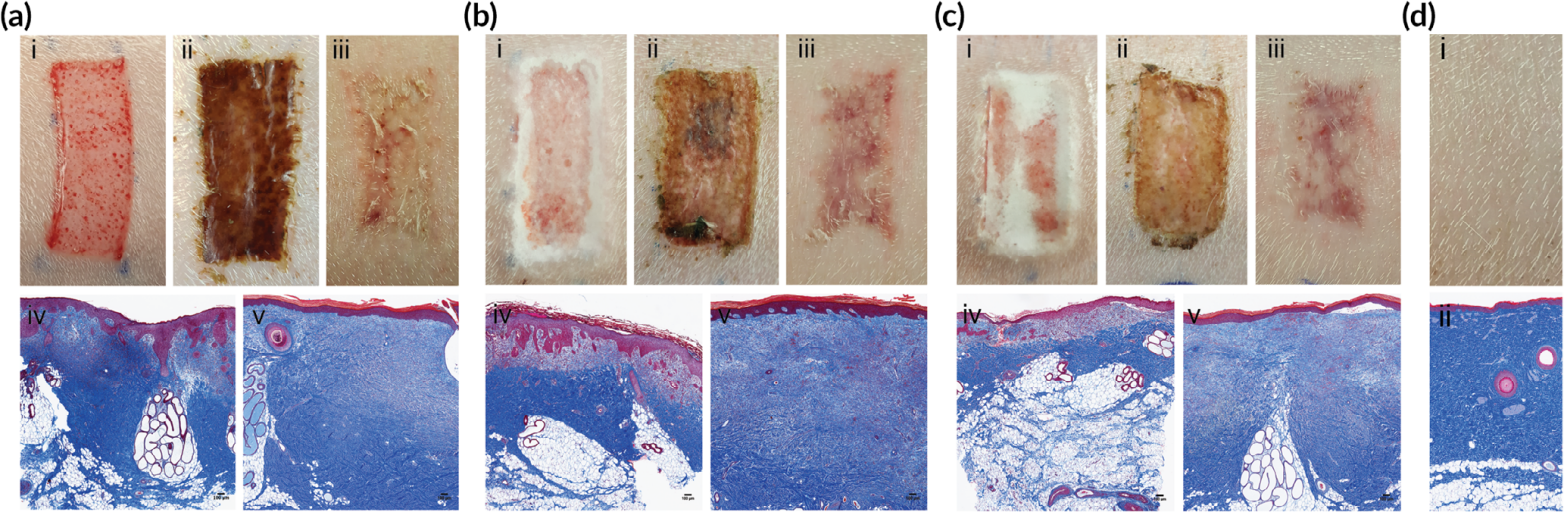
Partial‐thickness wounds dressed with Tegaderm (a), PLGA/PEG (b), PLGA/PEG/Ag (c). Wounds are pictured immediately after creation (i), after 7 days (ii), and after 35 days (iii). Masson's trichrome stained histology of biopsies taken after 7 days (iv) and 35 days (v). Healthy, unwounded skin is pictured (i) and shown in histology stained with Masson's trichrome (ii) for comparison (d)

Partial‐thickness wound healing is shown in Figure 6. Wounds approximately 600 μm deep were created and immediately dressed with either PLGA/PEG, PLGA/PEG/Ag, or Tegaderm (6). PLGA/PEG and PLGA/PEG/Ag are deposited as fibers that, at body temperature, transition to become adhesive and conformal to the wound bed (Figure 6bi,ci). Compared to the polymer dressed wounds, scabs that develop over the Tegaderm‐dressed wounds appeared darker (6). Shedding of the scab began by the second week of wound healing with a shiny new layer of epidermis visible by PWD 14 on all wounds. At PWD 35, all wounds were healed with complete dermal regeneration and re‐epithelialization (Figure 6). For comparison, healthy, unwounded skin is shown in Figure 6d. No infections or other significant complications developed in any of the wounds.

### 2.6 Dermal and epidermal tissue regeneration

**Figure 7:**
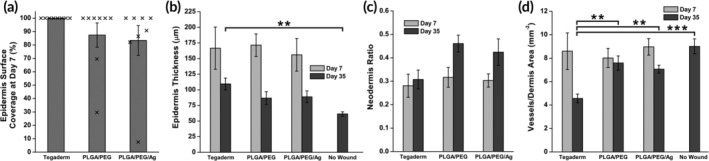
Histological characteristics of partial‐thickness wound healing for PLGA/PEG, PLGA/PEG/Ag, and Tegaderm wound dressings on PWD 7 and 35 (*n* = 8). (a) Average surface coverage of the epidermis on the healing wound biopsied at PWD 7. Individual data points are overlaid. (b) Epidermis thickness of the healing wounds. (c) Ratio of neodermis thickness to total dermis thickness. (d) Blood vessel density in the dermis of the healing wound. Asterisks indicate statistical significance: *p < .05; **p < .01; *** = *p* < .001

Wound healing was assessed by histology, which showed only slight differences in healing rates between the dressing groups. On PWD 7, Tegaderm‐dressed wounds had complete epidermis coverage across the surface of the biopsy on histology (Figure 7a). Two PLGA/PEG and four PLGA/PEG/Ag dressed wounds showed incomplete epidermal regrowth. However, total epidermal thicknesses were similar across the dressing groups on PWD 7 (Figure 7b). At PWD 35, epidermis thickness was similar between PLGA/PEG‐dressed wounds and healthy, unwounded skin, while Tegaderm was significantly thicker (Figure 7b).

After injury, the regenerating dermis (neodermis) consists of loose collagen fibers that are remodeled into dense, mature bundles as the wound heals. Stained with Masson's trichrome, these collagen structures appear light blue, while uninjured, organized collagen fibrils appear dark blue. The thickness of the neodermis was compared to the total thickness of the dermis, to determine how much neodermis was deposited relative to total dermis thickness (Figure 7c). On PWD 7, the neodermis ratio was similar among all wounds. On PWD 35, wounds dressed with Tegaderm had the lowest neodermis ratio. Angiogenesis is an important factor in the early stages of wound healing that can be measured by the dermis blood vessel density.^37^ Despite initially being similar at PWD 7, blood vessel density is significantly increased at PWD 35 for PLGA/PEG, PLGA/PEG/Ag, and the no wound control when compared to Tegaderm (Figure 7d). All wounds have decreased blood vessel density compared to unwounded skin at PWD 35.

**Figure 8:**
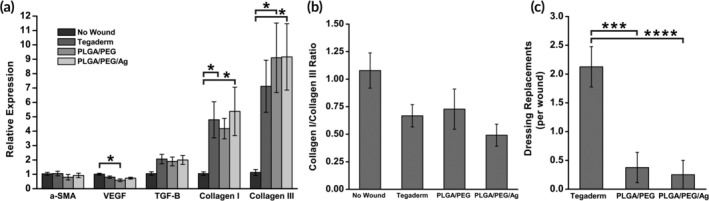
(a) RT‐PCR measurements of wound healing genes α‐smooth muscle actin (α‐SMA), vascular endothelial growth factor (VEGF), transforming growth factor‐β1 (TGF‐β1), collagen I, and collagen III on PWD 35 (*n* = 8). Gene expression measured relative to those of normal uninjured skin. (b) Ratio of collagen I expression to collagen III expression. (c) Number of required dressing replacements per wound due to dressing deadherence (*n* = 8). Asterisks indicate statistical significance: *p < .05; **p < .01; ***p < .001

The gene expression of critical factors in wound healing and scarring was measured in the wound tissue at PWD 35. Expression levels of α‐smooth muscle actin (α‐SMA), vascular endothelial growth factor (VEGF), transforming growth factor‐β1 (TGF‐β1), collagen type I, and collagen type III were measured using RT‐PCR relative to the normal, unwounded skin. Overall, there were no statistically significant differences between the wounds dressed with Tegaderm, PLGA/PEG, and PLGA/PEG/Ag (Figure 8a). Collagen I to collagen III ratio, which is indicative of how much disorganized, scar‐forming collagen is deposited, is also similar between dressings (Figure 8b).

Wound dressings must be replaced if exudate buildup causes the potential for maceration, or if the dressing comes off the wound. Over the first 14 days of wound healing, dressings were assessed for maintaining adherence to the wound bed. Wounds were checked daily, and dressings were replaced as needed. At PWD 14, all dressings were removed, and all wounds showed a visible layer of epidermis. Tegaderm dressings required a replacement at a significantly higher frequency than PLGA/PEG or PLGA/PEG/Ag (Figure 8c). Tegaderm dressings rely on a pressure sensitive adhesive coating to maintain adherence, but normal movement and fluid accumulation resulted in separation and loss of dressing. As the wound heals, the SBS dressings become less flexible but adhere strongly as they are integrated into the scab, which contains exudate and blood. Ultimately, some PLGA/PEG is degraded, while other parts may be displaced by the eschar as the wound epithelializes and come off with the scab.

## 3 DISCUSSION

An ideal dressing is easy and painless to apply, antimicrobial, keeps a moist wound environment, and requires minimal dressing changes while still protecting the wound. Here, we demonstrate that SBS allows for in situ sprayable wound dressing deposition with minimal wound contact. This ensures consistency with “no‐touch” technique, which is used in clinical practice to minimize transfer of infectious microorganisms and protect the wound.^38^ A portable airbrush can deliver the initially fibrous sealant (Figure 1) directly onto the wound bed. A biodegradable PLGA/PEG blend provides inherent adhesion and occlusion, integrating into the wound and reducing the rate of dressing replacement. Adding AgNO_3_ to the PLGA/PEG fibers gives the wound dressing antimicrobial capacity (Figure 4a) that does not compromise its mechanical properties (Figure 3a–c), thermal characteristics (Figure 3d), tissue adhesion strength (Figure 3e), or biocompatibility (Figure 4b).

The benefits of a moist wound environment provided by an occlusive dressing are established, with faster re‐epithelialization and less scarring.^39^, ^40^, ^41^ Dry, solid polymer wound dressings such as Tegaderm provide a semipermeable barrier to pathogens and liquid flow, allowing air and water vapor in the wound to be exchanged. Tegaderm was thus chosen as the clinical control. Alternatives such as autologous keratinocyte grafts and bilayered skin substitutes, which incorporate living cells to improve wound healing, may also be used in combination.^42^, ^43^ However, synthetic options that incorporate both an effective scaffold for wound healing and a solid barrier to pathogens and liquid transport are limited. Hydrogel and silicone‐based multilayered dressings have been developed to provide both adequate hydration and an internal layer of synthetic matrix, often composed of polyurethane or nylon.^44^, ^45^ These devices are typically only used selectively due to difficulty of use or high cost.^46^, ^47^

PLGA/PEG and PLGA/PEG/Ag dressings adhere to the wound and absorb exudate, forming a durable polymer‐scab hybrid scaffold that protects the wound and falls off after re‐epithelialization and keratinization of the wound bed. PLGA/PEG and PLGA/PEG/Ag dressings can be easily applied and require no dressing changes until the wound is healed (Figure 5a). When fabricated with fluorescent PLGA, PLGA molecules are present in the regenerated dermis (Figure 5b), indicating its absorbability and the close interface formed that allows for re‐epithelialization and collagen regeneration (Figure 5e). Unlike the thin film polyurethane dressings, the SBS dressings showed high adhesiveness to the raw wound bed and required fewer replacements (Figure 8c). This study further supports existing evidence that biodegradable polyesters—although relatively uncommon in wound dressings—can produce excellent results as scalable, low cost alternatives for large TBSA wounds.^48^, ^49^

Wound healing is a complex and tightly regulated inflammatory process, during which excessive inflammation leads to greater scaring.^50^ Skin injury produces a cascade of events that includes infiltration of neutrophils, fibroblasts, and endothelial cells.^51^ In a milieu of inflammatory cytokines and growth factors, new components of the dermis and epidermis are laid down throughout the stages of wound healing. All wounds healed at similar rates and with similar cosmetic results in terms of scarring (Figure 6a–c). While the Tegaderm dressing was found to have more complete epidermal surface coverage on wound biopsies on PWD 7 (Figure 7a), the slower rate of re‐epithelialization with the PLGA/PEG/Ag dressings is not surprising given the known inhibitory effects of silver on keratinocyte, fibroblasts, and epidermal cells^52^, ^53^ and did not have a negative effect on outcomes. Future studies could probe the antimicrobial efficacy of PLGA/PEG/Ag in vivo with an infection challenge model or by quantifying the bacterial burden in wounds over time.^54^, ^55^

The PLGA/PEG dressed wounds showed similar dermal and epidermal thicknesses at PWD 7 (Figure 7b) to wounds covered with thin‐film polyurethane‐based Tegaderm, indicating that dermal tissue regeneration was not affected by PLGA/PEG. Further, at PWD 35, epidermal thickness for PLGA/PEG and PLGA/PEG/Ag was 47 and 43% closer, respectively, to healthy skin than that of Tegaderm. Significant epidermal hypertrophy indicates greater levels of inflammation during the early stages of wound healing.^56^ Although similar at PWD 7, at PWD 35, blood vessel density is significantly increased for PLGA/PEG and PLGA/PEG/Ag compared to Tegaderm (Figure 7d). This indicates greater levels of angiogenesis in the healing dermis during the proliferative stage of wound repair which is critical for healing.^57^, ^58^ Revascularization could be stimulated by the lactic acid supplied by the biodegradable PLGA scaffold, which has been shown to increase angiogenesis.^59^, ^60^, ^61^

High expression of TGF‐β leads to excess fibroblast activity, which contributes to the development of hypertrophic scars and keloids. The composition of collagen subtypes can also give insight into the degree of scarring that may ultimately develop in the wounds. Scarless healing has been linked to greater collagen III deposition, while hypertrophic and keloidal scars have increased collagen production and an increased ratio of collagen I to collagen III.^62^ At PWD 35, there were no significant differences in the expression of these factors in the wounds treated with the three dressing groups (Figure 8a), indicating that all wounds are past the inflammatory phase of wound healing. All wounds had greater expression of collagen III than collagen I (Figure 8b), likely indicating similar amounts of scarring.

Pigs, which provide the best animal model for human skin,^63^ expose the wounds to unpredictable trauma and shear forces. Tegaderm dressed wounds suffered significantly greater dressing loss than those dressed with PLGA/PEG or PLGA/PEG/Ag (Figure 8c). The most likely reason is the moist environment, which reduces the adhesiveness of the dressing. Ultimately, PLGA/PEG and PLGA/PEG/Ag have better adhesion to wounds because they form a durable protective interface with tissue that is reinforced with fibrin from coagulated blood and can absorb exudate. However, shear forces from animal movement and cage trauma led to some dressing retraction from the wound edges. This likely created a drier and less protected environment at the wound edge, delaying the rate of re‐epithelialization seen at the biopsy sites.

## 4 MATERIALS AND METHODS

### 4.1 Solution blow spinning process and polymer solutions

A commercially available airbrush (Master Airbrush G22‐SET, 0.2 mm nozzle diameter) was used in all SBS protocols involved in the following experiments. For porcine in vivo wound healing studies, a handheld, CO_2_ cartridge‐fed regulator was used. In all other studies, gas was supplied through a CO_2_ tank equipped with a regulator. The distance between the airbrush nozzle and the application surface was approximately 10 cm for all studies. All polymer solutions were dissolved in ethyl acetate (Fisher), except for those that were dissolved in acetone (Fisher) for morphology studies using the scanning electron microscope. Polymer solutions consisted of 10% w/v PLGA (inherent viscosity = 0.86 dL g^−1^ in hexafluoroisopropanol, M_n_ = 48,800 ± 450 g mol^−1^ measured with gel permeation chromatography against Agilent polystyrene standards, 50:50, Lactel), and 5% w/v PEG (M_n_ = 950–1,050 g mol^−1^, MilliporeSigma). AgNO_3_, (MilliporeSigma) if added, was added after polymer dissolution and the solution was stirred overnight before use.

### 4.2 Scanning electron microscopy and energy‐dispersive X‐ray spectroscopy

A Hitachi SU‐70 Schottky field emission gun scanning electron microscope was used to image nanofiber mats sputter coated with gold. Snapshots were taken across the surface of the fiber mat. Fiber diameter and porosity were determined using the DiameterJ plugin for ImageJ (*n* = 2–4).^64^ EDS was used to measure weight fraction. EDS‐determined abundances were converted to weight fraction based on the three primary elemental components of the polymer fibers being carbon, oxygen, and silver.

### 4.3 Tensile mechanical testing

Tensile tests were made using a TA Instruments DMA Q800 equipped with a film tension clamp. Samples were stretched under a controlled force ramp from 0 to 5 N at a rate of 0.01 N min^−1^. Measurements were made either at room temperature or at 37°C after a 10 min isothermal period. Elastic modulus was calculated from the linear region of the resulting stress/strain curve. Each sample type was replicated five times (*n* = 5).

### 4.4 Differential scanning calorimetry

Approximately 10 mg samples of fiber mats were sealed in aluminum hermetic pans (TA Instruments) using a sample encapsulation press. Differential scanning calorimetry (DSC) measurements were made on a TA Instruments DSC Q100. Samples were held isothermal at −50°C for 5 min and then heated and cooled from −50 to 80 to −50°C, at a rate of 10°C min^−1^ for two continuous cycles. The inflection point of the heat flow during the T_g_ was used to determine the midpoint.

### 4.5 Silver release studies

A 2 mL of polymer solution was used to fabricate fiber mats containing various amounts of AgNO_3_, which were then weighed (*n* = 3). The fiber mats were then submerged in 4 mL of deionized water, and stored at a constant temperature of 37°C. The supernatant was sampled periodically over 30 days. Samples were analyzed by ICP‐AES on a Shimadzu ICPE‐9000, measuring at 328 nm for Ag^+^. Mass of silver released was normalized to the mass of the fiber mat.

### 4.6 Wound closure adhesion testing

Wound closure adhesion testing was performed on the TA Instruments DMA Q800. 1 cm by 1 cm sections of porcine skin were attached to rectangular clamps using cyanoacrylate glue. The rectangular clamps were brought together end to end, and 1 mL of sealant polymer solution was deposited on this joint, closing the gap between the two skin‐coated clamps (see ASTM F2458–05).^65^ The sealant was carefully applied and trimmed to avoid coating the interface between the ends and edges of the clamps. It was then allowed to set at 37°C for 10 min before testing. A controlled force ramp was used to increase force at a rate of 1 N min^−1^ until failure. Failure type was recorded as either adhesive or cohesive. Force values were normalized to the surface area of skin coated by the adhesive, which was measured using calipers, giving adhesive strength. Each sample type was replicated five times (*n* = 5).

### 4.7 Antimicrobial zone of inhibition

The antibacterial effects of the PLGA/PEG/Ag dressing were tested in vivo against *Staphylococcus aureus* (ATCC 6538) and *Escherichia coli* (ATCC 8739) using standard disk susceptibility testing methods.^66^, ^67^ PLGA/PEG/Ag antimicrobial effects were tested against a broad‐spectrum antibiotic, gentamicin sulfate (MilliporeSigma). Blank 10 mm Kirby–Bauer disks (MilliporeSigma) were loaded with gentamicin solution in sterile water at a potency of 10 μg.^68^ After air‐drying for 15 min, the gentamicin disks were pressed onto the agar. Disks of PLGA/PEG/Ag (1 mg/mL, Ag‐M, and 5 mg/mL, Ag‐H) were made by spraying 5 mL of PLGA/PEG/Ag solution onto sterile glass coverslips and cutting into 5 mm disks (*n* = 3–5). The disks were pressed onto the agar surface with at least 2 cm distance between disks. All disks were then wet with 30 μL sterile water to facilitate release of Ag^+^. After incubation for 24 hr at 37°C, the ZOI for each disk was measured using a caliper. ZOI was normalized to the disk diameter.

### 4.8 Mouse L929 fibroblast cytotoxicity

Cytotoxicity of the SBS polymer dressings was tested against L929 mouse fibroblasts by elution method as described by ISO‐10993‐5.^69^ PLGA/PEG/Ag and PLGA/PEG were blowspun onto sterile 5 × 5 cm coverslips. The polymer mats were then removed from the coverslips and eluted at mass concentration of 10 mg/mL in culture media of Dulbecco's modified Eagle medium supplemented with 10% fetal bovine serum (Gemini Bio‐Products Inc.), L‐glutamine and 1% penicillin and streptomycin at standard conditions (37°C, 5% CO_2_) for 24 hr. The elutions were then diluted to 1×, 10×, and 100× dilutions, and cell viability was tested against the different dilutions.

L929 fibroblasts (10^5^ cells/mL) were then plated into 96‐well plates at 100uL per well and incubated for 24 hr under standard conditions. The culture media was then removed by pipette. Wells were then treated to control (standard media), 25 ug/mL puromycin, or diluted elutions of PLGA/PEG/Ag and PLGA/PEG. This measurement was repeated eight times for each diluted elution (*n* = 8).

### 4.9 Porcine partial‐thickness wound healing model

Animal studies were performed in the research animal facility at Children's National Health System with IACUC approval (protocol #30454). Two 20–25 kg Yorkshire swine were used in this pilot study. Six partial‐thickness (0.6 mm depth setting) skin wounds were made on each side of the paravertebral skin with a dermatome (Humeca), making a total of 12 wounds per animal. The wounds were made 1.5 cm long in cranial‐caudal direction and 4 cm wide. Each wound was separated by 1.5 cm of normal skin. The wounds were randomized to treatment by Tegaderm (3 M), PLGA/PEG, or PLGA/PEG/Ag, resulting in a total of four wounds per dressing group per animal, over two animals (*n* = 8). Wounds were uniformly sprayed with 2 mL of polymer solution, which produced complete wound coverage as determined by a surgeon. Wounds were assessed daily for healing and signs of infection by visual inspection. Dressing replacement was performed as needed until PWD 14. The experimental endpoint was chosen to be 35 days after initial wound creation. Wound healing was followed by wound size and scar tissue measurements by caliper on PWD 3, 7, and 35.5 mm full thickness punch biopsies of the wounds were also taken on PWD 7 and 35. Biopsies on PWD 7 were taken 1 cm from the lower left corner of the wound, while biopsies for PWD 35 were taken at the center of the wounds.

### 4.10 Histological analysis

Biopsied tissues were kept in 10% neutral buffered formalin until histological processing (Histoserv Inc.). Punch biopsy samples were bisected along the longitudinal axis then embedded in paraffin wax. 5 μm sections were prepared and fixed onto glass slides and then stained with Masson's trichrome. Digital images of the histology slides were taken with TissueScope LE (Huron Digital Pathology) at 40× magnification then exported for analysis with ImageJ. Images were scaled to 1 μm/pixel. Epidermal and dermal thicknesses were measured after cropping images to 3,000 μm by 3,000 μm. Epidermis thickness was measured at areas that show at least a basal layer of epidermal cells, stained red in Masson's trichrome. Total dermis thickness was measured from the base of the epidermis to the level of subdermal fat. Also measured was the thickness of the evolving dermal matrix, seen as disorganized collagen bundles (in light blue) above the layer of organized collagen bundles (dark blue). Thickness measurements were taken at the left, middle, and right third of the images, and then averaged. The vascular density (vessels per mm^2^ of dermis) of each biopsy was measured by counting the number of unique vessel structures in the dermis, including arterioles and venules, but not capillaries. Dermis area was measured using ImageJ. Density measurements were made by two researchers and averaged.

### 4.11 Wound healing gene expression

Gene expression of α‐SMA, VEGF, TGF‐β1, collagen I and collagen III in the healed wounds were quantified using real‐time PCR (RT‐PCR). Full thickness biopsies were taken from the center of the healed wounds at PWD 35. Normal uninjured skin biopsies were also taken from both sides of the paraspinal back skin with samples from the upper and lower back. Gene expressions in the wounds were measured relative to those expressed in normal skin tissue. Biopsied tissues were snap‐frozen in liquid nitrogen, and then stored at −80°C until analysis.

RNA extraction from frozen tissue was performed by tissue homogenization in Trizol reagent (Life Technologies) and PureLink RNA Mini Kit (Thermo Fisher Scientific). For all experiments, 3 μg RNA was used to synthesize first stand cDNA using High‐Capacity cDNA Reverse Transcription kit (Life Technologies). RT‐PCR was performed using TaqMan® Gene Expression Master Mix (Life Technologies) in a QuantStudio 7 Flex RT‐PCR System (Thermo Fisher Scientific), according to the manufacturer's instructions. Reactions were performed in triplicate, including no template controls and amplification of a housekeeping gene, GAPDH. Gene‐specific assays were Ss03373340_m1 for COL1A1, Ss04245588_m1 for α‐SMA, Ss04323768_g1 for COL3A1, Ss03382325_u1 for TGF‐β1, and Ss03375629_u1 for GAPDH (Life Technologies). Changes in relative gene expression normalized to GAPDH levels were determined using the ΔΔCt method. The difference between the Ct values (ΔCt) of the gene of interest and the housekeeping gene is calculated for each experimental sample. Then, the difference in the ΔCt values between the experimental and unwounded skin samples ΔΔCt is calculated. The fold‐change in expression of the gene of interest between the two samples is then equal to 2^(−ΔΔ)^.

### 4.12 Statistical analysis

Statistical analysis was performed on Stata (StataCorp) or Origin (OriginLab). Typically, one‐way ANOVA was used to compare group variation, followed by post hoc pairwise Tukey comparison to determine significant differences between the groups. This procedure was not used for Figure 7a because the data sets are not normally distributed. Statistical significance is considered for *p* < .05. Typically, averages were plotted with error bars representing standard error. If no asterisks are shown, there are no significant differences among the groups.

## 5 CONCLUSIONS

Clinically, most partial thickness wounds would be dressed with antimicrobial ointments or dressings, like bacitracin or silver sulfadiazine, and gauze. These dressings are not occlusive and require daily dressing changes. They are often time‐consuming and difficult to apply, and painful for the patient. By controllably releasing AgNO_3_ from solution blow spun PLGA/PEG/Ag, the requirements for a rapid, broad‐spectrum antibiotic treatment and a highly adhesive wound dressing that absorbs exudate are met. Release of Ag^+^ can be tailored for lasting antimicrobial activity without incurring high cytotoxicity. The sprayable SBS process allows for simple application of a conformal dressing for wounds of any shape and size that does not need to be removed or changed. Overall, the use of PLGA/PEG‐based dressings is simple, effective, and was without significant wound complication or delay in healing in a porcine partial thickness wound model.

## CONFLICT OF INTEREST

The authors have no conflicts of interest.

## Supporting information


**Figure S1** Estimated wound concentration plotted and fit with a second order logarithmic model.
**Figure S2**. PWD 7 histology slides with vessels indicated (black arrows). Black shapes outline nondermis areas.
**Figure S3**. More PWD 7 histology slides with vessels indicated (black arrows).
**Figure S4**. PWD 35 histology slides with vessels indicated (black arrows).
**Figure S5**. More PWD 35 histology slides with vessels indicated (black arrows).
**Figure S6**. Unwounded histology slides with vessels indicated (black arrows). Wounds are sorted by wound location.
