# Supplementary material for: Sprayable and biodegradable, intrinsically adhesive wound dressing with antimicrobial properties
Source: Bioeng Transl Med. 2019 Dec 13;5(1):e10149. doi: 10.1002/btm2.10149 (PMC6971445; doi:10.1002/btm2.10149)
Supplement: Supplementary file 1 — Figure S1 Estimated wound concentration plotted and fit with a second order logarithmic model. Figure S2. PWD 7 histology slides with vessels indicated (black arrows). Black shapes outline nondermis areas. Figure S3. More PWD 7 histology slides with vessels indicated (black arrows). Figure S4. PWD 35 histology slides with vessels indicated (black arrows). Figure S5. More PWD 35 histology slides with vessels indicated (black arrows). Figure S6. Unwounded histology slides with vessels indicated (black arrows). Wounds are sorted by wound location. [file BTM2-5-e10149-s001.doc]

**Supplementary Information**

Sprayable and Biodegradable, Intrinsically Adhesive Wound Dressing with Antimicrobial Properties

*John L. Daristotle^†^, Lung W. Lau^†^, Metecan Erdi, Joseph Hunter, Albert Djoum Jr., Priya Srinivasan, Xiaofang Wu, Mousumi Basu, Omar B. Ayyub, Anthony D. Sandler, and Peter Kofinas^*^*

**Results**

*Wound Silver Concentration Model:* In vitro silver release data (μmol g^–1^) from Figure 3A was used as the basis for this model. The data from Figure 3A (which is normalized for wound dressing mass) was first multiplied by the expected weight of a single wound dressing: 0.3 g based on spraying 2 mL of 15% w/v polymer solution. Concentration (μM) was determined by dividing by the expected wound volume for an average sized wound in the partial thickness wound model. The average wound area was 8 ± 1 cm^2^ based on PWD 0 wound measurements. The penetration depth of Ag^+^ into the wound was estimated to be 7.5 mm, based on reference [38].

MATLAB Curve Fitting Toolbox was used to fit this adjusted concentration (*I*) versus time (*t*) data from Figure 3A. A second order logarithmic model with general format was used:


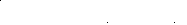


The results are shown in **Figure S1**. A logarithmic model was created to enable the following differential equation to be solved, where *k_A_* = 0.2434 day^–1^ is the first order rate constant combining absorption, distribution, metabolism, and excretion in this skin, per reference [39].


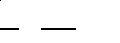


This differential equation was solved over 28 days and plotted in Figure 3C for each AgNO_3_ concentration in solution that was investigated.


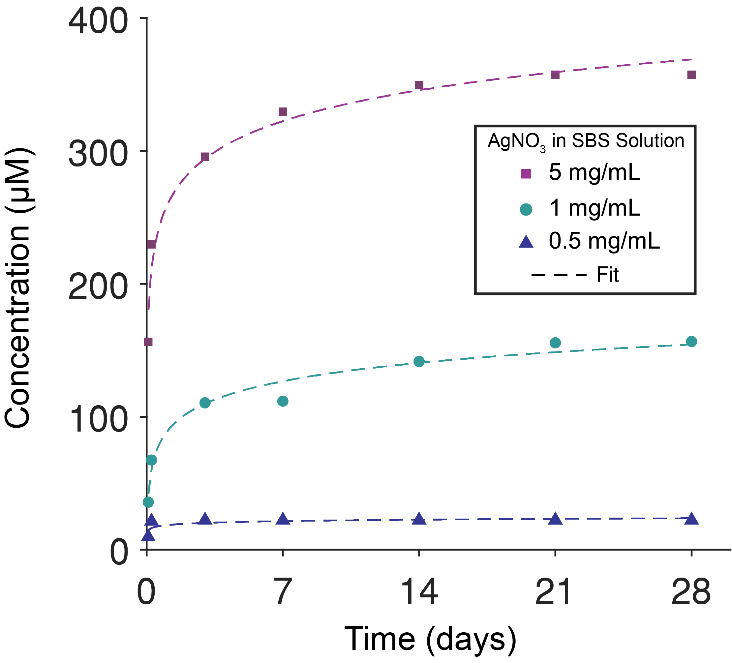


**Figure S1**. Estimated wound concentration plotted and fit with a second order logarithmic model.

*Blood Vessel Count*

The vascular density (vessels per mm^2^ of dermis) of each biopsy was measured by counting the number of unique vessel structures in the dermis, including arterioles and venules, but not capillaries. Sections from PWD 7, PWD 35, and unwounded skin are shown below. Vessels on each section were labeled with a black arrow. Non-dermis areas (including glands and hair follicles) were excluded from the dermis area calculation, which was made using ImageJ.


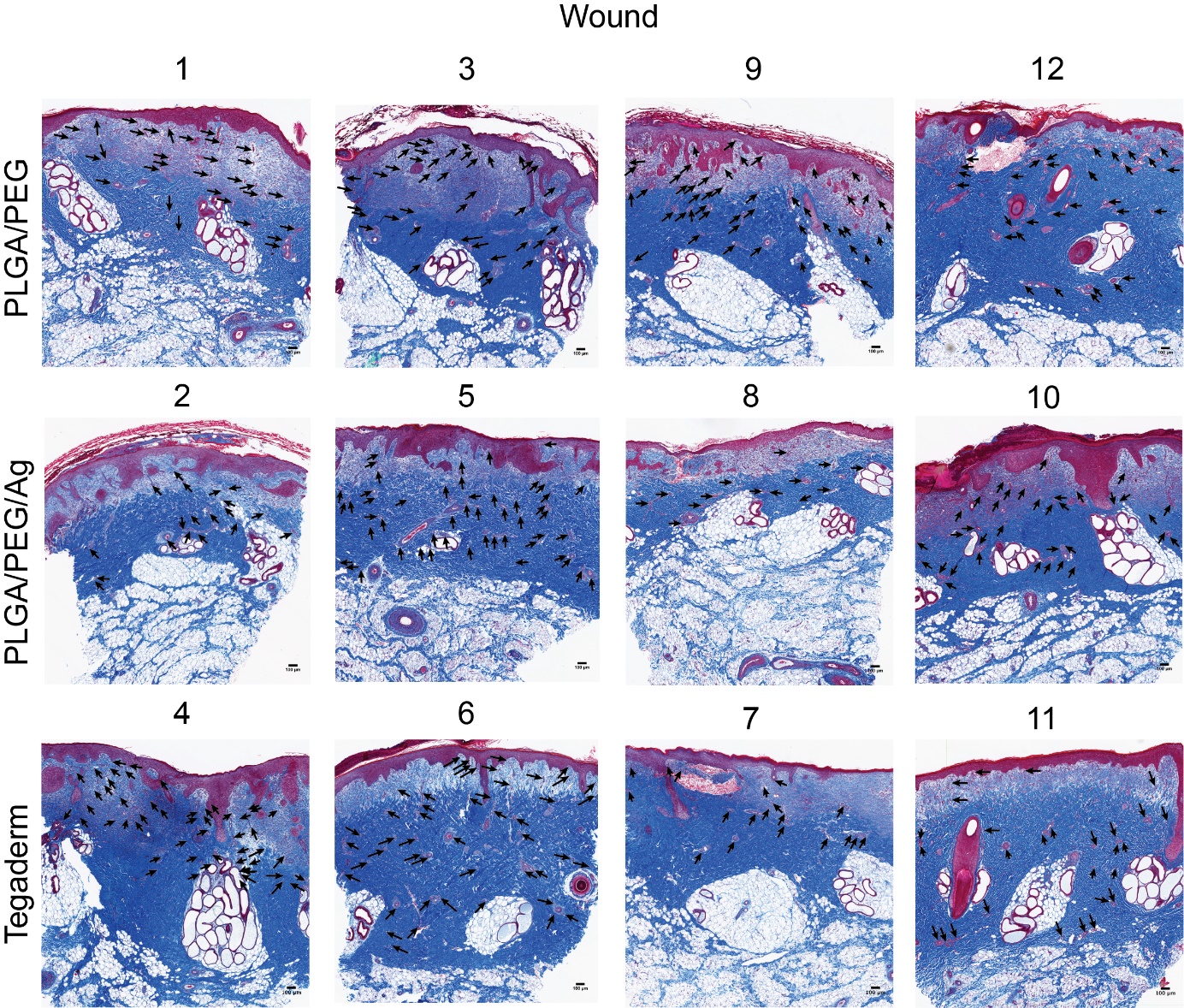


**Figure S2**. PWD 7 histology slides with vessels indicated (black arrows). Black shapes outline non-dermis areas.


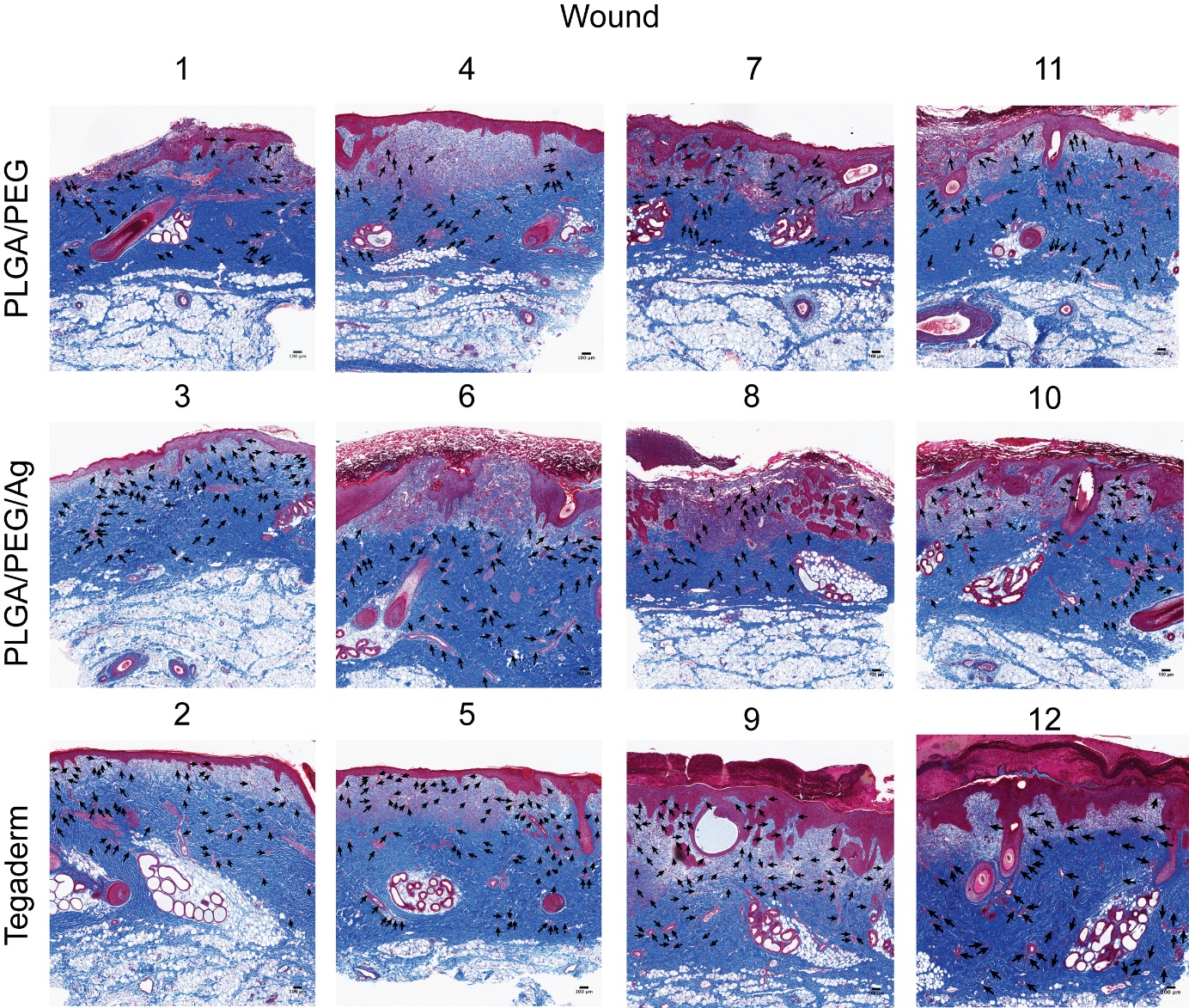


**Figure S3**. More PWD 7 histology slides with vessels indicated (black arrows).


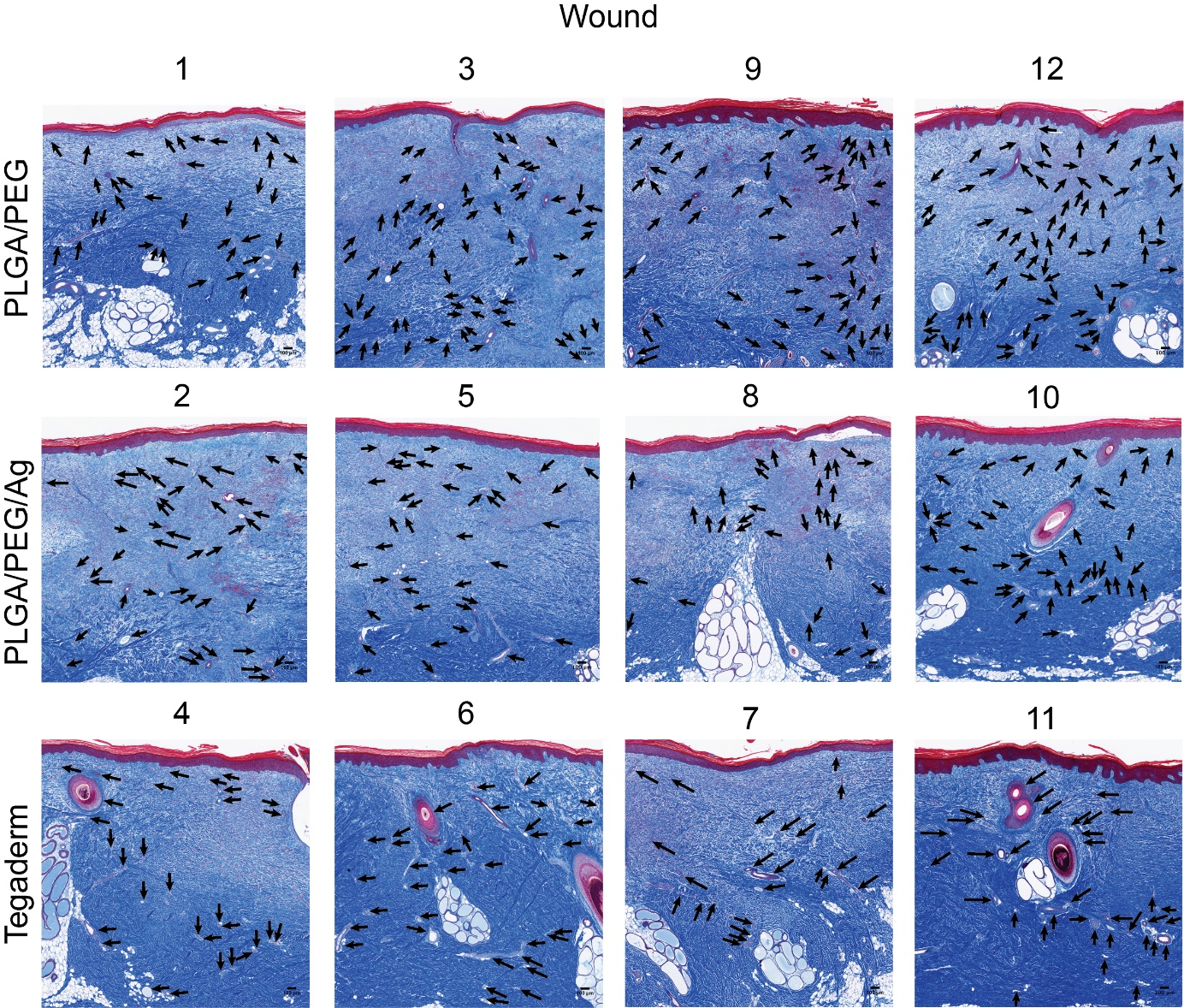


**Figure S4**. PWD 35 histology slides with vessels indicated (black arrows).


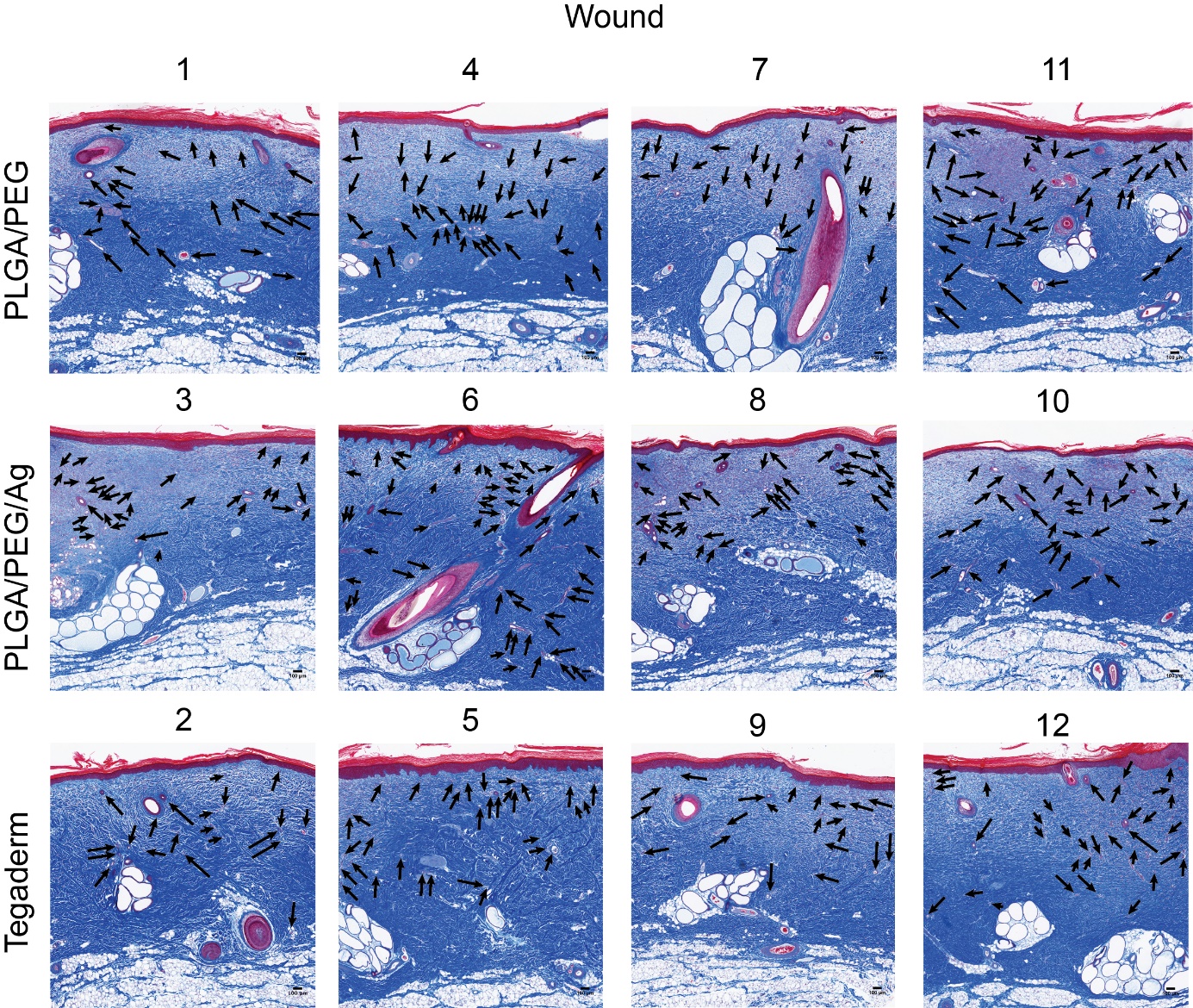


**Figure S5**. More PWD 35 histology slides with vessels indicated (black arrows).


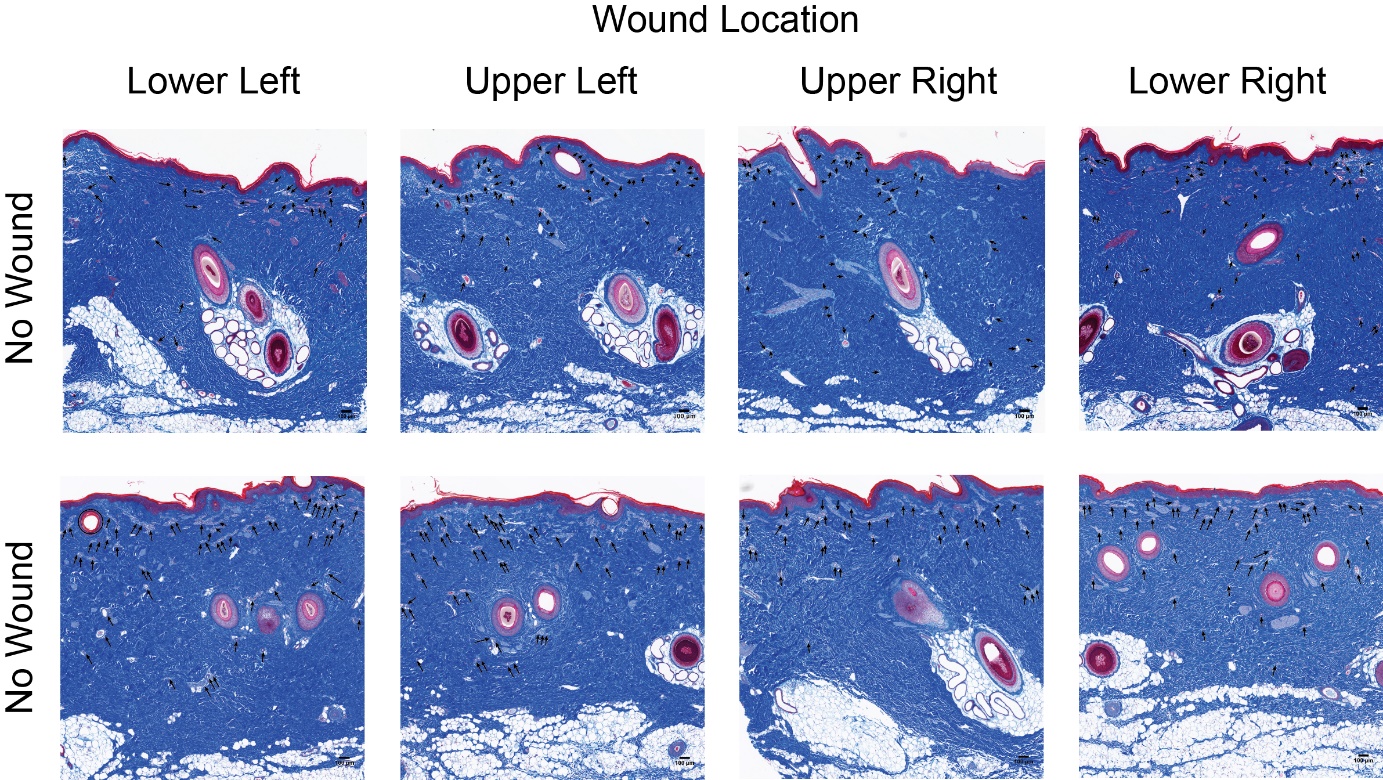


**Figure S6**. Unwounded histology slides with vessels indicated (black arrows). Wounds are sorted by wound location.
